# Surgical thoracic duct decompression: The right choice for Fontan-associated protein-losing enteropathy?

**DOI:** 10.1016/j.xjtc.2025.04.013

**Published:** 2025-05-03

**Authors:** Mohamad Alaeddine, Deepti P. Bhat, Joshua Pohlman, Joseph Graziano, Vasu Gooty, Hüseyin Sicim, Daniel A. Velez

**Affiliations:** Cardiothoracic Surgery and Fontan Clinic, Phoenix Children's Hospital, and University of Arizona, Phoenix, Ariz

**Keywords:** protein losing enteropathy, Fontan patients, surgical thoracic duct decompression

## Abstract

**Objective:**

Protein-losing enteropathy is among the most debilitating complications of Fontan circulation. Central venous hypertension increases lymphatic pressure in the thoracic duct, potentially leading to significant intestinal protein loss. Current treatment options for recurrent or refractory protein-losing enteropathy are limited to complex lymphatic interventions, fenestration creation, and heart transplantation. We have implemented an alternative surgical approach—thoracic duct decompression—and report our early experience in pediatric Fontan patients.

**Methods:**

We studied 5 Fontan patients, analyzing their preoperative history, treatment, and surgical approach tailored to their unique anatomy, postoperative course, and symptoms at their most recent Fontan clinic follow-up.

**Results:**

Since February 2024, 5 Fontan patients aged 5 to 16 years diagnosed with recurrent protein-losing enteropathy underwent surgical thoracic duct decompression. The first 2 patients experienced mild anastomotic narrowing, which was successfully treated with transcatheter angioplasty. The remaining 3 patients had uneventful postoperative course and hospital stays of approximately 1 week. All patients were closely monitored with echocardiograms and laboratory testing. At 6 months postoperatively, all remained symptom-free and transplant-free (follow-up range, 7-12 months), and reported improved quality of life. No cases of turndown stenosis were observed. Additionally, all patients were successfully weaned off enteral steroids and aggressive diuretic therapy. A mild decrease in oxygen saturations (1%-3%) was noted with no clinical significance.

**Conclusions:**

Thoracic duct decompression appears to be a feasible intervention for recurrent protein-losing enteropathy in Fontan patients, demonstrating a low rate of short-term complications. This procedure may serve as a viable alternative to heart transplantation in select cases; however, its long-term efficacy warrants further investigation.

**Figure:**
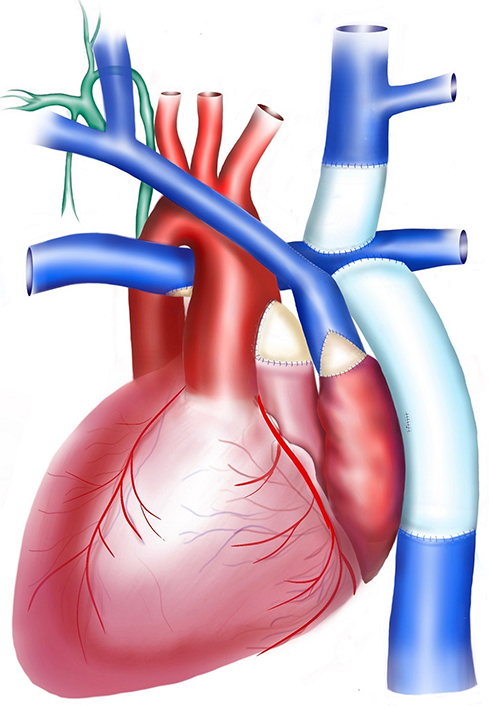
Thoracic duct decompression is performed by rerouting the venous drainage to the heart.

**Central Message**

Thoracic duct decompression procedure is a viable option to treat protein-losing enteropathy in select patients with Fontan physiology.

**Perspective**

Based on our experience and short-term results, we believe that thoracic duct decompression should be the first choice for treatment of medically refractory protein losing enteropathy. There will be failures and recurrences in some, but this procedure will not burn bridges for further lymphatic interventions.

The Fontan procedure is the final stage of palliation for patients with functionally univentricular circulation, achieved by creating a total cavopulmonary anastomosis. In this circulation, systemic venous pressure must be sufficiently elevated to enable passive lung perfusion, compensating for the absence of right ventricular pumping.^1^^,^^2^ As an unintended consequence, increased lymphatic production and outflow venous pressure lead to elevated thoracic duct pressure. Some patients with Fontan circulation therefore develop chronic, recurrent, and debilitating lymphatic complications, most notably protein-losing enteropathy (PLE).^3^ Although the etiology of PLE is multifactorial, a subset of cases has been linked to thoracic duct congestion and elevated pressure. This pressure is transmitted back to the intestinal lymphatics, causing dilation, lacteal rupture, and protein leakage into the intestine.^1^, ^2^, ^3^

PLE can affect 3% to 18% of Fontan patients, often resulting in repeated hospitalizations, intravenous protein replacement, and recurrence despite maximal medical therapy, including aggressive diuresis, pulmonary vasodilators, and corticosteroids, all of which carry their own adverse effects.^3^ Historically, refractory and recurrent PLE has prompted referral for heart transplantation as the only viable option. However, these patients remain at high risk for transplant-related mortality and PLE may often recur even after a successful heart transplantation.^4^

Experimental evidence has suggested that redirecting thoracic duct drainage into a pulmonary vein or the atrium may alleviate PLE.^1^^,^^5^ This proposed lymphatic-venous right-to-left shunt aims to reduce venous outflow pressure on the intestinal lymphatic system, thereby decreasing protein and lymph loss. However, the technical feasibility of this procedure in patients with complex univentricular anatomy remains largely untested with limited case reports of thoracic duct decompression (TDD) via a transcatheter approach^6^ and surgical approach.^7^^,^^8^

We report our institutional experience with surgical TDD in pediatric Fontan patients with recurrent and refractory PLE. We detail the surgical techniques used to accommodate the individual anatomic variation and describe outcomes.

## Methods

This retrospective study was conducted at Phoenix Children's Hospital and approved by the institutional review board (IRB-20-374; approved April 27, 2021). A waiver of consent was granted for retrospective use of the clinical data.

The study population included all pediatric Fontan patients diagnosed with severe and recurrent PLE who were evaluated by our multidisciplinary clinic and referred for TDD as a treatment for PLE. All surgical procedures were performed between February 2024 and July 2024. All patients had their diagnosis of PLE confirmed with stool alpha-1-antitrypsin (AAT) levels. We routinely monitored the serum albumin levels during follow-up, but only repeated the stool AAT levels if there were any laboratory or clinical markers for recurrence of PLE.

Data collected included demographic characteristics, type of single-ventricle anatomy, prior surgical procedures, onset, and treatment of PLE before the TDD. Additional data included intraoperative details and immediate postoperative short-term outcomes, such as the need for reintervention, readmission for PLE, transplantation, or mortality. Follow-up data included clinical metrics (oxygen saturations, laboratory markers, and PLE-related symptoms), subjective exercise tolerance, and caregiver- or patient-reported quality of life. Imaging data confirming the status of TDD anastomosis was also collected during follow-up.

## Results

Table 1 summarizes the clinical and hemodynamic characteristics of the study population. The age at operation ranged from 5 to 16 years. Patients 3 and 5 had heterotaxy syndrome and dextrocardia. Venous anomalies were present in 3 patients: bilateral superior vena cavae (SVCs) in patient 1 and 2, left sided SVC in patient 5, and total anomalous pulmonary venous connection in patient 3. Cardiac transplant was considered as an option for many of these patients before referral. Patient 1 was not considered to be a suitable candidate due to multiorgan dysfunction and social concerns related to medication compliance. Patient 3 was offered a transplant evaluation, but the family elected to pursue all alternative options before considering transplant. Patient 5 declined cardiac transplantation evaluation.

**Table 1:** Presurgical imaging and hemodynamic data of the patients who underwent thoracic duct decompression

Case	Age (y)	Cardiac diagnosis with significant extracardiac comorbidity	Preoperative (hemodynamic data)	Echocardiogram assessment (pre-surgical)	PLE onset after Fontan and therapy
1	9	DILV with bilateral SVC, pulmonary stenosis - PLE after a History of multiorgan failure with septic shock, renal failure requiring dialysis, ECMO support, diabetes	- Mean Fontan pressure ∼16-17 mm Hg - SVEDP = 8 mm Hg - ASD restriction (MG = 5-6 mm Hg) - PVR = 3.7 WU - Patent Fontan pathway - Diminished cardiac function	- Patent Glenn and Fontan pathway - Trivial atrioventricular valve regurgitation - Normal single LV function	3 y Prolonged admission with albumin infusions. - PDE5 inhibitor - Enteral steroids - Diuretics (furosemide or spironolactone) - SGLT2i - ACE inhibitor
2	5	Tricuspid atresia with L-TGA, pulmonary stenosis - COVID-19, colitis before surgery	- Mean Fontan pressure ∼16 mm Hg - SVEDP = 5 mm Hg - ASD restriction (MG = 5-6 mm Hg) - PVR = 2.3 WU - Saturation = 86% - Qp:Qs = 1:1 - Patent Fontan pathway - Prominent AP collaterals	- Restrictive atrial shunt (MG ∼8-10 mm Hg) - Normal single LV function - Patent Fontan pathway	7 mo Prolonged admission for fluid management - PDE5 inhibitor - Spironolactone - Enteral steroids - Diuretics (furosemide or spironolactone) - Serial albumin infusions
3	12	Heterotaxy, dextrocardia, right IVC, bilateral SVC, right atrial isomerism, TAPVR, right dominant common atrioventricular valve with TGA, right aortic arch	- Mean Fontan pressure ∼16-17 mm Hg - SVEDP = 11 mm Hg - PVR = 1.8 WU - Saturation = 95% - Qp:Qs = 1:1 - Patent Fontan pathway, prominent AP collaterals coiled	- Mild-moderate AV valve regurgitation - Normal single LV function - Patent Fontan pathway	4 y - Recurrent admissions, underwent extensive lymphatic interventions - PDE5 inhibitor - Enteral steroids - Diuretics (furosemide, spironolactone, or hydrochorthiazide) - Weekly albumin infusions
4	7	Levocardia, unbalanced RV dominant atrioventricular canal, coarctation. Normal systemic and pulmonary venous anatomy Myhre syndrome G-tube dependent	- Mean Fontan pressure ∼18 mm Hg - SVEDP = 13 mm Hg - Ascending aorta to transverse aorta gradient ∼20 mm Hg - PVR = 1.5 WU - Saturation = 85% - Patent fenestration Qp:Qs = 0.9:1	- Mean transpulmonary gradient ∼4 mm Hg - Moderate common AV valve regurgitation - Normal systemic LV function	8 mo - Recurrent admission for intravenous albumin infusion and intravenous diuretics - PDE5 inhibitor - Diuretics (furosemide or spironolactone) - Enteral steroids
5	16	Heterotaxy, dextrocardia, double-outlet right ventricle with D-malposed great arteries, left sided SVC	- Mean Fontan pressure ∼12 mm Hg - SVEDP = 9 mm Hg - PVR = 1.3 WU - Saturation = 96% - Qp:Qs = 1:1 - Patent Fontan pathway	- Trace AV valve regurgitation - Normal single ventricle function	1 y - Recurrent admission for intravenous albumin infusion and intravenous diuretics - PDE5 inhibitor - Diuretics (furosemide or spironolactone) - Enteral steroids - Midodrine - SGLT2i

*PLE*, Protein-losing enteropathy; *DILV*, double inlet left ventricle; *SVC*, superior vena cava; *ECMO*, extracorporeal membrane oxygenation; *SVEDP*, systemic ventricle end-diastolic pressure; *PDE5*, phosphodiesterase type 5 inhibitor; *ASD*, atrial septal defect; *MG*, mean gradient; *PVR*, pulmonary vascular resistance; *LV*, left ventricle; *SGLT2i*, sodium-glucose cotransporter-2 inhibitors; *ACE*, angiotensin-converting enzyme; *L-TGA*, levo-transposition of great arteries; *AP*, aorto-pulmonary; *IVC*, inferior vena cava; *TAPVR*, total anomalous pulmonary venous return; *RV*, right ventricle.

### Preoperative Assessment for Surgical Candidacy for TDD

The diagnosis of PLE was confirmed with fecal AAT levels. All patients had AAT levels >113 mg/dL (normal value for the laboratory, ≤54 mg/dL), with patient 3 demonstrating AAT levels 575 mg/dL at diagnosis. All patients underwent cardiac workup, including a hemodynamic cardiac catheterization, echocardiographic assessment, and magnetic resonance lymphangiogram (MRL) to confirm the patency and drainage location of the thoracic duct. Dynamic contrast-enhanced MRL was performed on 3T scanners with direct injection of dilute gadoterate meglumine contrast into the bilateral inguinal lymph nodes. Precontrast T2-weighted imaging was also performed. Mesenteric and supraclavicular soft tissue edema-like signal was identified for all patients, with patient 3 demonstrating the most pronounced signal abnormalities and patient 1 showing relatively mild edema. No patient had significant perihilar edema. All patients also demonstrated a patent thoracic duct without notable ectasia. The termination of the thoracic duct was visualized at 4.5, 2.5, 6, 6.5, and 15 minutes for patients 1 through 5 after the initiation of contrast administration. All thoracic ducts terminated at the confluence of the subclavian and internal jugular veins, but on the left for patients 1, 2, and 4, and on the right for patients 3 and 5. Table 2 shows the details of the pertinent MRL findings for each patient. Marked lymphatic reflux occurred into the mesenteric root with eventual pooling of contrast in the duodenum for patient 3. Patient 1 had mild lymphatic reflux into the mesenteric root but no bowel accumulation. No supraclavicular or superior mediastinal lymphatic reflux was visualized for patient 1. Patients 2 through 5 demonstrated contrast reflux into the supraclavicular soft tissue with extension into the superior mediastinum next to the termination of the thoracic duct.

**Table 2:** Surgical details and postsurgical course of Fontan patients who underwent surgical thoracic duct decompression (TDD) for recurrent protein-losing enteropathy

Case	Age (y)	Prior surgical procedures	Dynamic contrast-enhanced MR lymphangiography	Surgical technique for TDD	Post-TDD course Postoperative LOS	Assessment at last follow-up
1	9	- BTT shunt (3 d) - Bilateral bidirectional Glenn (4 m) - Non-fenestrated EC Fontan (18 mm): 6 y	- Mild mesenteric and supraclavicular edema. - TD patent, termination visualized at 4.5 min to the left venous angle - Mild lymphatic reflux into the mesenteric root. No reflux into the supraclavicular soft tissues	Takedown of left-sided Glenn LSVC to RA appendage anastomosis	- Stenosis of turndown anastomosis requiring stent angioplasty about 3 wk after surgery - 5 wk	- 12 mo postoperatively - No clinical recurrence - Normal albumin levels (3.5 g/dL) - Patent anastomosis by echocardiogram and CT angiography at follow-up - SPo_2_ change (90% preop, 87% at follow-up) - Patient lives at ∼7000 feet elevation
2	5	- Atrial septectomy, DKS, bidirectional Glenn (6 m) - Non-fenestrated EC Fontan (18 mm); (4 y)	- Moderate mesenteric and supraclavicular edema - TD patent, termination visualized at 2.5 min to the left venous angle - Moderate lymphatic reflux into the supraclavicular soft tissues and superior mediastinum, but none into the mesenteric root	Placement of 8 mm interposition (reinforced) graft from innominate vein to RA appendage; revision of EC Fontan (18 mm, non-fenestrated interposition graft), atrial septectomy, unroofing of coronary sinus	- Stenosis of turndown anastomosis requiring stent angioplasty (1 wk after surgery) - 5 wk	- 9 mo postoperatively - No clinical recurrence - Weaned off steroids and diuretics - Normal albumin levels (4.5 g/dL) - Patent anastomosis by echocardiogram and CT angiography - SPo_2_ change (94% preop, 94% at follow-up). - Patient reports improved energy, improved PO feeds, improved quality of life
3	12	- Bilateral, bidirectional Glenn (5 m) - Chemical pleurodhesis and attempted TD ligation (7 m) - Fenestrated EC Fontan, 18 mm (6 y)	- Marked mesenteric and moderate supraclavicular edema - TD patent, termination visualized at 6 min at the right venous angle - Marked lymphatic reflux into the mesenteric root and eventual contrast accumulation in the duodenum - Lymphatic reflux also present into the supraclavicular soft tissues	Takedown of right sided Glenn; and right SVC to RA appendage	- None - 1 wk	- 7 mo postoperatively - No clinical recurrence-until 5 mo when she developed a viral infection, causing severe ventricular dysfunction and severe common AV valve regurgitation. Subsequent cardiac catheterization showed severely elevated SVEDP ∼18-19 mm Hg in the setting of heart failure - Patent anastomosis by CT angiogram and echocardiogram - SPo_2_ change (93% preoperatively, 92% at follow-up at 5 mo)
4	7	- Coarctation repair, PA banding (1 m) - DKS, right sided bidirectional Glenn, pulmonary debanding (4 m) - Fenestrated, EC Fontan, 18 mm (6 y)	- Moderate mesenteric and supraclavicular edema - TD patent, termination visualized at 6.5 min to the left venous angle - Mild lymphatic reflux into the supraclavicular soft tissues and superior mediastinum, but none into the mesenteric root	- TDD with innominate vein turndown to appendage, aortic arch reconstruction	- Left vocal cord palsy - 1 wk	- 7 mo postoperatively - No clinical recurrence - Weaned off steroids after 5 mo - Normal albumin levels (4.4 g/dL) - Patent anastomosis by CT angiogram at discharge and echocardiogram at follow-up - SPo_2_ change (88% preoperatively, 87% at follow-up) - Patient reports improved energy, improved PO feeds, improved quality of life
5	16	- DKS, BTT shunt (1 m) - Bidirectional Glenn (4 m) - Fenestrated EC Fontan (4 y)	- Moderate mesenteric and supraclavicular edema with thickened duodenal wall - TD patent, termination visualized by 15 min to the right venous angle. - Mild lymphatic reflux into the supraclavicular soft tissues and superior mediastinum, but none into the mesenteric root	- TDD with innominate vein anastomosis to RA appendage, interposition graft from right IJV to RPA	- None - 1 wk	- 7 mo - No clinical recurrence - Normal albumin levels (4.4 g/dL) - Patent anastomosis by CT angiogram at discharge and echocardiogram at follow-up - SPo_2_ change (96% preoperatively, 93% at follow-up)

*MR*, Magnetic resonance; *LOS*, length of stay; *BTT*, Blalock-Thomas-Taussig shunt; *EC*, extracardiac; *TD*, thoracic duct; *LSVC*, left superior vena cava; *RA*, right atrial; *CT*, computed tomography; *Sp**o*_*2*_, peripheral oxygen saturation; *DKS*, Damus-Kaye-Stansel anastomosis; *PO*, postoperative; *SVC*, superior vena cava; *AV*, aortic valve; *SVEDP*, single ventricle end-diastolic pressure; *PA*, pulmonary artery; *IJV*, internal jugular vein; *RPA*, right pulmonary artery.

### Surgical Techniques

The procedure was tailored to the individual patient's anatomy. The procedure successfully resolved PLE in all patients (Table 2).

#### Case 1

A 9-year-old boy with double inlet left ventricle, bilateral SVCs with left juxtaposition of atrial appendages. His surgical history included bilateral bidirectional Glenn and nonfenestrated extracardiac Fontan. The thoracic duct was noted to drain to the left SVC venous system. The surgical approach included redo median sternotomy, aortic, bilateral SVC, and inferior vena cava (IVC) cannulation. The left SVC was detached from the left pulmonary artery (LPA) and anastomosed to the left juxtaposed right atrial appendage using a side-biting vascular clamp.

#### Case 2

A 5-year-old boy with tricuspid atresia (type 3), a single right-sided SVC, and a thoracic duct draining into the innominate vein system. His surgical history included atrial septectomy, Damus-Kaye-Stansel (DKS) anastomosis, bidirectional Glenn, and an 18-mm nonfenestrated extracardiac Fontan procedure. He presented with an early-onset PLE, restricted atrial septum, and further complications from COVID-19 colitis. The procedure involved redo median sternotomy, aortic and bicaval cannulation, and transection of the innominate vein off the right brachiocephalic vein junction. Using the dunk technique, an 8-mm ring-reinforced Gore-Tex (W. L. Gore & Associates) tube graft was used to bridge the innominate vein and the right atrial appendage.^6^

#### Case 3

A 12-year-old girl with heterotaxy, dextrocardia, right atrial isomerism, right ventricle-dominant unbalanced atrioventricular septal defect, malposed great arteries, right-sided IVC, and bilateral SVCs. Surgical history included bilateral bidirectional Glenn and 18-mm nonfenestrated extracardiac Fontan completion. She had an extensive pre-TDD history, including recurrent chylothorax post-Glenn operation, for which she had required pleurodesis and attempted thoracic duct ligation via right thoracotomy. After the onset of PLE, she underwent lymphatic workup and was referred to an outside institute for lymphatic intervention involving embolization of mesenteric leaks. However, the transcatheter procedure was unsuccessful with recurrence of her symptoms and PLE within 4 to 6 weeks. Preoperative evaluation revealed that the thoracic duct was intact and drained to the right SVC venous system. The surgical approach included redo median sternotomy, aortic, and IVC cannulation. Detachment of the right SVC and anastomosis to the adjacent common atrial appendage using a side-biting vascular clamp. Albumin levels normalized within 1 week.

#### Case 4

A 7-year-old girl with Myhre syndrome, right ventricle dominant unbalanced atrioventricular septal defect, and aortic coarctation. Surgical history included DKS, arch repair, bidirectional Glenn, and later 18-mm extracardiac Fontan. She presented with an early onset recurrent PLE and midaortic arch gradient. The thoracic duct drained to the innominate vein. Surgery involved redo median sternotomy, innominate artery, SVC, right atrial appendage cannulation, aortic arch patch augmentation, and innominate vein turndown to the left atrial appendage. She was discharged home in 1 week.

#### Case 5

**Figure 1:**
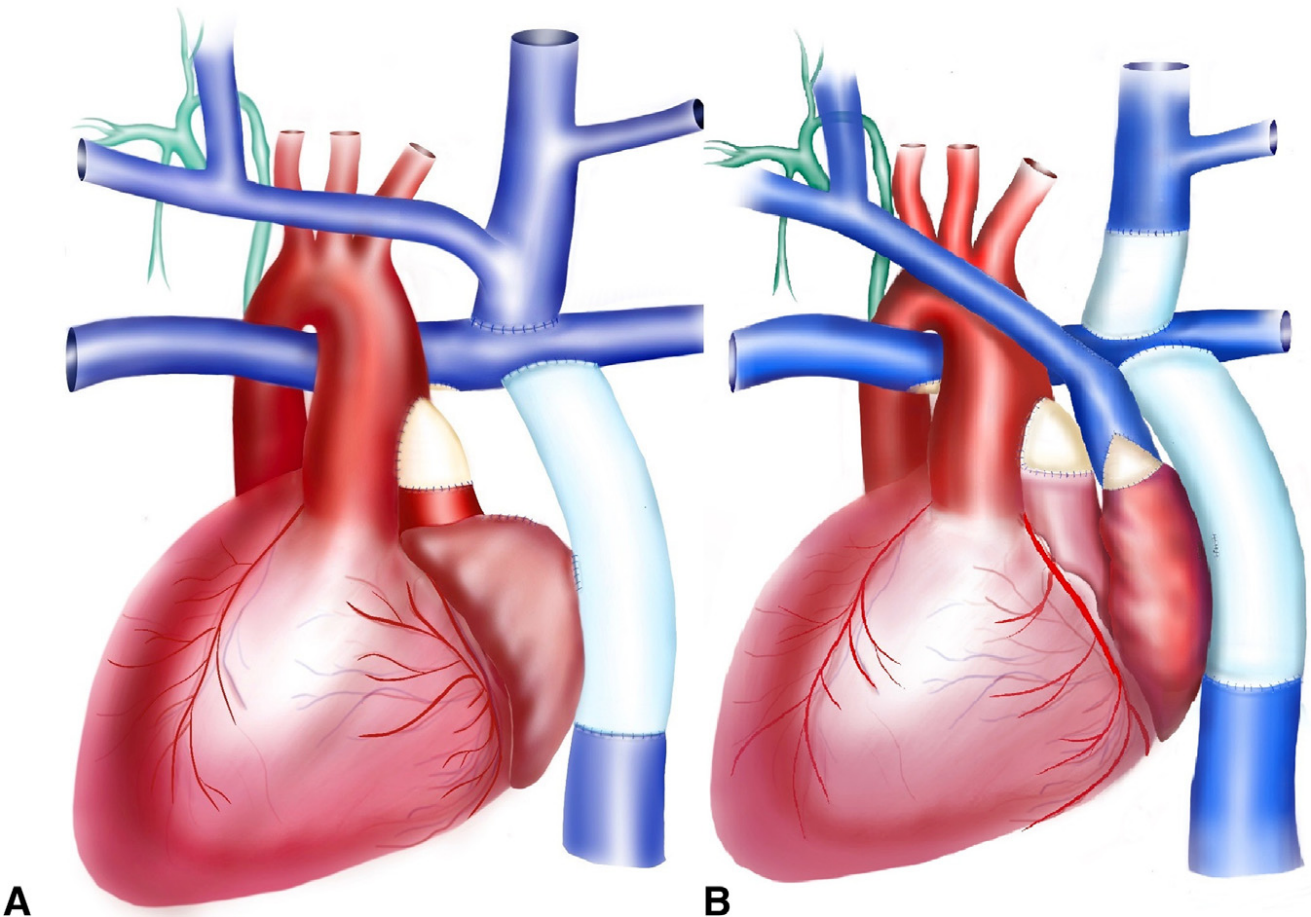
Illustration of anatomy. A, Mitral atresia, double outlet right ventricle, situs inversus dextrocardia, status-post extracardiac fenestrated Fontan with thoracic duct draining to the right side (*in green*), and surgical decompression. B, Takedown of the fenestration, division of the left internal jugular vein (*LIJV*) and transection of the left-sided superior vena cava (*SVC*). Placement of interposition graft between the LIJV and the left pulmonary artery. The decompression was completed with anastomosis between the SVC and the left-sided right atrial appendage. The anterior-lateral aspect of the anastomosis was augmented using a bovine pericardial patch.

A 16-year-old with heterotaxy, situs inversus, mitral atresia, double-outlet right ventricle, left-sided SVC and IVC. Surgical history included pulmonary artery band, DKS, and Blalock-Thomas-Taussig shunt. This was followed by a left-sided bidirectional Glenn procedure. The patient then underwent ventricular septal defect enlargement and extracardiac fenestrated Fontan. The thoracic duct drained to the right internal jugular, subclavian vein junction. Surgical approach included redo sternotomy, aortic, SVC, and IVC cannulation. The Fontan fenestration was taken down and the left-sided atrial mass was mobilized. The left-sided internal jugular was transected at the SVC junction. The distal end was oversewn, and the SVC was detached from the LPA. A 14-mm Gore-Tex interposition graft was placed between the left internal jugular and the LPA. The SVC was then anastomosed to the left-sided right atrial appendage. The anterior-left aspect of the anastomosis was enlarged using a bovine pericardial patch (Figure 1). The patient did well after surgery and was discharged home in 1 week.

### Anticoagulation Regimen After Surgery

All patients were treated with aspirin prophylaxis before surgery. Patients 1 and 2 were maintained on aspirin postoperatively but developed mild anastomosis stenosis. Both underwent successful stent angioplasty of the turndown anastomosis before discharge and were sent home on a direct-acting oral anticoagulant, namely rivaroxaban. Patients 3 and 5, both of whom had a nonfenestrated Fontan, received aspirin before TDD. Their postoperative hospital course and uneventful, and they were discharged on aspirin and alone, which they continue at follow-up. Patient 4 underwent a fenestrated Fontan completion with aortic arch reconstruction at the time of TDD. She was maintained on 6 weeks of dual antiplatelet therapy (aspirin and clopidogrel) and subsequently maintained on aspirin alone.

### Postoperative and Short-Term Outcomes

Table 2 provides a detailed summary of each patient's clinical course before and after surgical TDD procedure. All patients were on maximal medical management before referral to surgery. At the time of manuscript preparation, the minimum follow-up duration was 7 months. There was 100% survival free of transplant and symptoms at follow-up. None of the patients reported any neurological complications in the immediate postoperative period or reported any neurological symptoms at the time of follow up.

Patient 1 had a prolonged hospitalization before surgery due to refractory PLE following streptococcal sepsis. After TDD, he continued to exhibit low serum albumin levels, and a cardiac computed tomography angiogram showed stenosis of the surgical turndown anastomosis. He subsequently underwent successful stent angioplasty of the turndown site, which resulted in the resolution of PLE symptoms within 1 week after the stent angioplasty. At his most recent follow-up (12 months postoperatively), he remained symptom-free with a serum albumin >3.5 g/dL. Of note, this patient resides at an elevation or approximately 7000 feet and has ongoing comorbidities, including diabetes mellitus and pulmonary hypertension, for which he is receiving pulmonary vasodilator therapy.

Patient 2 also experienced a prolonged hospitalization before surgery, with PLE exacerbation by COVID-19 colitis. Post-TDD, he demonstrated persistently low serum albumin levels, and a computed tomography angiogram obtained within the first postoperative week showed narrowing at the turndown anastomosis. He underwent stent angioplasty with a resolution of his PLE symptoms within 1 week after stent placement. At his last follow-up at 9 months, his oxygen saturation ranged from 93% to 95% with patent turndown anastomosis by computed tomography scan. His serum albumin level exceeded 4 g/dL, and his mother reported a substantial improvement in his energy level and quality of life.

Patient 3 had undergone extensive lymphatic intervention before TDD surgery, including lymphatic coiling and embolization. She experienced resolution of PLE symptoms within 1 week of surgery and remained well without clinical or laboratory evidence of PLE for 5 months. However, she then developed a viral illness followed by progressive single ventricle dysfunction and severe atrioventricular valve regurgitation. Initially managed with outpatient heart failure therapy, she was hospitalized 6 months post-TDD due to worsening symptoms. A computed tomography angiogram during this admission confirmed a patent TDD anastomosis, but cardiac catheterization revealed severely elevated end-diastolic pressures (18-19 mm Hg) and markedly depressed ventricular systolic function. Her serum albumin levels had declined to <2 g/dL, requiring intensive diuretic therapy.

Patient 4 had an uneventful postoperative recovery and was discharged home 1 week after surgery. Following TDD, her oral feeding and weight gain improved significantly, and she demonstrated increased energy levels. Her oxygen saturation stabilized at 88% on room air. She was successfully weaned off steroids and diuretics during follow-up.

Patient 5 also recovered well and was discharged home 1 week postoperatively. Her serum albumin levels remained between 2.2 and 2.5 g/dL for approximately 2 months, without the need for readmission or albumin replacement therapy. After 2 months, her albumin levels increased to >4 g/dL and has remained stable despite discontinuation of enteral steroid treatment.

All the patients continue to be followed closely in the multidisciplinary Fontan clinic, with monthly echocardiography, cross-sectional imaging, and laboratory testing to monitor for both clinical and subclinical signs of PLE recurrence. We routinely monitored serum albumin levels during follow-up, but only repeated the stool AAT levels if there were any laboratory or clinical markers for recurrence of PLE. We have not yet repeated cardiac catheterization on any of these patients for routine post-TDD assessment. Our plan is to repeat cardiac catheterization at about 1 year after the procedure for surveillance unless indicated.

## Discussion

PLE remains a severe and debilitating complication of Fontan circulation, often necessitating consideration of cardiac transplantation. We report our early experience with surgical TDD as an alternative surgical approach in our practice to alleviate thoracic duct congestion by redirecting lymphatic flow into the atrium. This approach aims to relieve PLE symptoms, improve quality of life, and provide an option free from both PLE and the need for cardiac transplantation.

Our experience demonstrates the technical feasibility of TDD in pediatric patients with unique and complex single-ventricle anatomy. We report complete resolution of PLE symptoms in all 5 patients and successful weaning from aggressive medical therapies, with minimal complication rates. This outcome was achieved through careful surgical planning and postoperative monitoring within the framework of a dedicated multidisciplinary Fontan team. Although limited data currently exist on the role of TDD in managing PLE, our series represents 1 of the largest single-center reports to date describing outcomes following surgical TDD in this patient population.^6^^,^^8^

A variation of the TDD procedure via the transcatheter route has been proposed as an alternative option by some authors^7^; however, experience with this technique remains limited to specialized centers. Although the transcatheter approach avoids surgery, it presents technical challenges and carries risks such as organ or endovascular perforation, stent migration, and potential need for surgical intervention. Given these considerations, our center favored the surgical approach, which we found to be more controlled, technically straightforward, and associated with fewer complications. Our experience demonstrates that this technique is feasible even in cases with complex anatomical variations.

Our center employed a highly selective, multidisciplinary approach in identifying surgical candidates. Inclusion was limited to patients with PLE and preserves single ventricle systolic function. There were no anatomic exclusions because the procedure was tailored to the individual patient's anatomy. We carefully reviewed the hemodynamic data and offered the procedure to patients with relatively normal single ventricular end-diastolic pressure (≤13 mm Hg), to allow the benefit of redirecting the lymphatic flow away from Fontan circulation to a relatively lower pressure atrial chamber. Notably, only patient 3 experienced recurrence of PLE. She had a preoperative TDD systemic ventricle end-diastolic pressure of approximately 13 mm Hg and later developed elevated systemic ventricle end-diastolic pressure (18 mm Hg) following a viral illness and ventricular decompensation, potentially reducing the effectiveness of the turndown procedure. Patients with Fontan-associated PLE have been reported to have overall lower mean pulmonary arterial pressure (<15 mm Hg) by some reports. However, 3 of our 5 patients had moderately elevated mean pulmonary arterial pressure (Fontan pressures) before the TDD. It is difficult to explain why some patients develop PLE and some do not despite having similar hemodynamics.^2^^,^^3^ Although it is believed to be multifactorial, our thought process is that, if there was no PLE before Fontan, then restoring the pre-Fontan outflow pressure might reverse it.

During this process, our team continues to learn. After our first patient was diagnosed with an obstruction to the turndown anastomosis after 3 weeks, we changed our surveillance practice with a predischarge routine cross-sectional study to confirm anastomosis patency and regular follow-up in the Fontan clinic. Our current follow-up includes repeat laboratory tests and echocardiogram within 1 month of discharge, and after weaning off PLE medications. In addition, we perform cross-sectional imaging every 6 months, or earlier if there are clinical concerns. Our patients did not have a significant drop in their oxygen saturation (1%-3%) nor did need any intervention to limit the degree of right to left shunt, such as banding the jugular vein above the thoracic duct entrance as described by some authors.^6^

Among the most important outcomes of this study was improvement in the perceived quality of life by our patients and their families. Freedom from recurrent symptoms, hospitalizations, missed school, and parent's time were some of the subjective benefits reported by families, although difficult to quantify, are critical to overall well-being.

We acknowledge the limitations of our study. The sample size is small, and follow-up duration was limited to maximum of 12 months. Nonetheless, this series adds to the very limited existing literature and supports the feasibility and short-term efficacy of surgical TDD in this challenging patient population.

## Conclusions

TDD is a technically feasible and well-tolerated surgical option for pediatric patients with Fontan-associated PLE. Careful preoperative evaluation, including assessment of cardiac systolic and diastolic function and anatomy of the lymphatic drainage, is essential for procedural planning. In our experience, TDD has a high rate of symptom-free and transplant-free survival at 6 to 12 months. Ongoing multidisciplinary surveillance remains vital to ensure long-term success. Future research with longer follow-up and expanded cohorts is necessary to confirm the reliability of this procedure as a viable alternative to cardiac transplantation.

### Webcast

You can watch a Webcast of this AATS meeting presentation by going to: Xxx.

## Conflict of Interest Statement

The authors reported no conflicts of interest.

The *Journal* policy requires editors and reviewers to disclose conflicts of interest and to decline handling or reviewing manuscripts for which they may have a conflict of interest. The editors and reviewers of this article have no conflicts of interest.
